# Co‐Design of Maternity Discussion Cards for Aboriginal and Torres Strait Islander Women

**DOI:** 10.1111/hex.70437

**Published:** 2025-09-19

**Authors:** Tanya Druce, Vidanka Vasilevski, Cara Kennedy, Ann Hallett, Karah Edwards, Linda Sweet, Debra Kerr

**Affiliations:** ^1^ School of Nursing & Midwifery, Centre for Quality & Patient Safety Research in the Institute for Health Transformation Deakin University Geelong Victoria Australia; ^2^ Western Health St Albans Victoria Australia

**Keywords:** Australian Aboriginal and Torres Strait Islander peoples, communication, decision‐making, first nations, pregnancy, shared

## Abstract

**Background:**

Aboriginal and Torres Strait Islander women and babies are more likely to have poorer health outcomes compared with non‐Indigenous women. Barriers exist for Aboriginal and Torres Strait Islander women to access high‐quality maternity care, and women can experience culturally unsafe care, racism and a lack of shared decision‐making.

**Aim:**

To develop maternity discussion cards to facilitate shared decision‐making between Aboriginal and Torres Strait Islander women and their partners/support persons, and their maternity care clinicians.

**Methods:**

Co‐design methodology in two workshops was used to develop the maternity discussion cards.

**Findings:**

Through a co‐design process, a set of 56 topic cards and three blank cards were developed. Additional cards, including an acknowledgement of country and a description of the artwork used on the cards, were designed, and guidelines for card use were also established. Strategies including Aboriginal‐led engaging activities, appropriate remuneration for Indigenous knowledge holders, and a culturally safe community setting were integral to the process of planning, designing and reviewing the maternity discussion cards.

**Discussion:**

It is envisaged that the cards will promote communication and shared decision‐making in the healthcare setting. The cards may provide women with an avenue for initiating difficult conversations. While developed in an urban setting, the cards could be adapted for digital use and tailored to rural and remote healthcare environments.

**Conclusion:**

A co‐design process enabled the development of a practical maternity resource for expectant families. The maternity discussion cards will be piloted in a maternity setting to be evaluated and further refined by end users.

**Patient or Public Contribution:**

Women who identified as Aboriginal or Torres Strait Islander or who had a baby who identified as Aboriginal or Torres Strait Islander were integral to co‐designing the maternity discussion cards. Their knowledge and feedback informed the design, content and guidelines for use of the maternity discussion cards.

## 1 Background

The perinatal mortality and morbidity rates are significantly higher for Aboriginal and Torres Strait Islander babies compared with non‐Indigenous babies [1]. The maternal mortality ratio is almost three times higher for Aboriginal and Torres Strait Islander women compared with non‐Indigenous Australian women, with 14.4 deaths per 100,000 women versus 5.1 deaths per 100,000 women giving birth, respectively [1, 2]. In addition, Aboriginal and Torres Strait Islander women have higher rates of preterm birth (before 37 weeks of gestation) and low birthweight babies (less than 2.5 kg) than non‐Indigenous Australian women, both associated with significant neonatal morbidity and mortality [1].

Studies have found that Aboriginal and Torres Strait Islander women can experience culturally unsafe practices and racism in healthcare settings, which impedes access and contributes to inequitable health outcomes [3, 4]. Evidence shows that Aboriginal and Torres Strait Islander women have fewer opportunities to make decisions about their care in comparison to non‐Indigenous women [2], which may also contribute to reduced access to optimal healthcare. There is growing recognition that enhancing self‐determination is an important consideration for improving healthcare access and health outcomes of Aboriginal and Torres Strait Islander peoples [5].

The Australian Korin Korin Balit‐Djak Aboriginal Health, Wellbeing and Safety Strategic Plan (2017–2027) [6] encompasses Aboriginal self‐determination as a core principle underpinning all actions and domains in health, well‐being and safety. The provision of culturally appropriate information and trauma‐informed maternity care aims to empower Aboriginal and Torres Strait Islander women to make informed decisions about their care [7]. Mechanisms to improve cultural safety, self‐determination, information provision and shared decision‐making in healthcare interactions with Aboriginal and Torres Strait Islander peoples are needed [8]. Individuals have identified that prompts for supporting discussions in healthcare settings empower them to ask questions and feel prepared for their appointments [9]. However, there are few culturally responsive tools to support shared decision‐making for Aboriginal and Torres Strait Islander peoples. One example is a recently developed culturally safe online shared decision‐making resource about general health and well‐being for Aboriginal and Torres Strait Islander peoples, which was found to be well received by consumers, although the authors recognise that further work is needed to embed this resource into routine clinical practice [10]. A recent scoping review showed both clinicians and consumers reported benefits of question prompt lists, such as increased consumer confidence, care satisfaction and reduced health‐related anxiety [11].

The concept of co‐design refers to research, policy and actions that are designed with the meaningful involvement of end‐users [12]. As a methodology, co‐design can be found in various bodies of literature and contexts, ranging from participatory research design, design thinking and public sector policy and innovation [13]. In the Australian context, co‐design approaches that work proactively with marginalised groups who are the subject of a policy or programme intervention have increasingly been used in the health field [14].

Using co‐design to develop question prompt lists is more likely to meet the health information needs of end‐users and work more effectively in practice [15]. The use of card games to facilitate conversations with patients in palliative care has been found to be a useful and effective approach to tackling uncomfortable topics of dying, death and end‐of‐life care [16]. Culturally appropriate discussion cards, co‐designed and developed for use in a palliative care setting to facilitate discussions between healthcare workers and Aboriginal and Torres Strait Islander peoples at end‐of‐life, have been developed and were the inspiration for this project [17]. The Dying To Talk Aboriginal and Torres Strait Islander Discussion Resource [17], while well‐used, has not been formally evaluated.

Co‐design studies are especially important for developing interventions with Aboriginal and Torres Strait Islander peoples to support community‐led approaches, evaluation and self‐determination [18]. The aim of this study was to co‐design maternity discussion cards with women and their support persons, as well as maternity care clinicians employed in a dedicated maternity care service for Aboriginal and Torres Strait Islander women in Victoria, Australia. The maternity discussion cards have been printed for a trial implementation phase. The co‐design process is reported in this paper.

## 2 Methods

### 2.1 Study Setting

The study site is a major healthcare provider to one of the fastest growing and most diverse regions in Victoria, Australia. The service catchment includes urban and semi‐rural areas and covers the Aboriginal traditional lands of the Wurundjeri, Boonwurrung, Bunurong and Wadawurrung people of the Kulin nation. Approximately 3% of the population in the region identifies as Aboriginal and/or Torres Strait Islander [19]. The study site includes an Aboriginal Health Unit and a tertiary maternity service recording over 6,500 births per year, with various models of maternity care, including hospital‐based midwifery and obstetric‐led care, Midwifery Group Practice (MGP), and a midwifery‐led homebirth programme. There is also a specific MGP for Aboriginal and Torres Strait Islander women or women who are giving birth to an Aboriginal and Torres Strait Islander baby [20]. This service comprises a small multidisciplinary team of 10 staff, including midwives, a Koori Maternity Service worker and a social worker who provide continuity of care across pregnancy, birth and the postpartum period. The team is committed to providing culturally safe and respectful care and works in close partnership with the health service's Aboriginal Health Unit. The primary midwife will attend labour and birth (if available), and obstetric services are provided by the obstetrician who is allocated duty on the day of presentation for birth. The dedicated MGP service supported 61 Aboriginal and Torres Strait Islander women and families in the preceding 12 months of this study (July 2023 to July 2024), with numbers in the service increasing each year since its inception in 2018.

### 2.2 Study Design and Data Collection

Participatory action research design [21] is an approach to research that seeks to situate power within the research process for those who are most affected by a programme. The participatory nature of participatory action research refers to the active involvement of end‐users and practitioners. The process is linked to action, which leads to the people affected having increased control over their lives [22].

Co‐design methods were used in this study [23] to collaborate with end‐users in designing maternity discussion cards. Cultural responsiveness informed the design of the study and the co‐design workshops. Aboriginal‐owned businesses were used for the venue, catering, graphic design and printing.

Two co‐design workshops of 120 min duration were conducted to develop the maternity discussion cards. The purpose of these workshops was to inform and review the content, language, presentation and use of the maternity discussion cards based on participants' needs, wishes and experiences. Workshop activities were guided by the LUMA System of Innovation [23] human‐centred design methods, which encompass three key design skills – looking, understanding and making. Four members of the research team had completed LUMA [23] training. Qualitative data were collected using methods guided by workshop activities, such as sticky notes, drawings and field notes taken by the research team. Analysis of data was undertaken between the two workshop sessions by members of the research team to refine ideas for presentation at the second workshop.

### 2.3 Participants

Women who were clients of the dedicated Aboriginal and Torres Strait Islander MGP were invited to participate through nomination by their primary midwife or Koori Maternity Service worker. Women who had experienced a major adverse pregnancy outcome (i.e., neonatal death or stillbirth) were not invited to participate. Staff were eligible to participate if they provided care to women attending the dedicated MGP in 2024. Eligible staff included midwives, Koori Maternity Service workers, Aboriginal and Torres Strait Islander health workers, obstetricians and social workers.

### 2.4 Recruitment

Pregnant women interested in participating in the study provided their contact details on a form that was given to the principal investigator (TD) for follow‐up. The women who had expressed interest in participating were contacted using the details they provided. Women were invited to bring a partner or support person to the co‐design workshop if they wished. The participants were provided with a Participant Information and Consent Form (PICF) by email.

An associate investigator (A.H.) emailed maternity care clinicians employed in the MGP and Aboriginal Health Unit with a brief overview of the study and the PICF. The maternity care clinicians interested in participating in the workshops responded by email.

### 2.5 Ethics

Ethics approval was obtained from the healthcare organisations' Low Risk Ethics Panel (HREC no. 103748) and an academic institution ethics committee (2024/HE000830). Strategies were implemented to ensure Indigenous knowledge, practices and innovations were respected, protected and maintained throughout the project. For example, women and support people were remunerated consistent with the healthcare organisations' policy of consumer engagement. This practice aligns with guidelines that Aboriginal and Torres Strait Islander people who contribute knowledge, practice innovations, skills and know‐how to a project should receive fair and equitable benefits [24].

## 3 Results

There were 22 participants in Workshop 1, including nine consumers and 13 maternity care clinicians. The research team (*n* = 5) who facilitated the workshop included one Aboriginal member. Forty‐three percent (*n* = 9) of the workshop participants (women, support people and maternity care clinicians) identified as an Aboriginal or Torres Strait Islander person.

Workshop 2 was attended by eight consumers and 11 maternity care clinicians and was facilitated by the same research team members. Five consumers attended both Workshop 1 and Workshop 2. Thirty‐seven percent (*n* = 8) of Workshop 2 participants (women, support people and maternity care clinicians) identified as an Aboriginal or Torres Strait Islander person. The Aboriginal and Torres Strait Islander status of consumer attendees is outlined in Table 1.

**Table 1:** Workshop consumer attendees.

	Aboriginal and Torres Strait Islander women	Non‐Indigenous women	Aboriginal and Torres Strait Islander support people	Non‐Indigenous support people	Total
Workshop 1	3	2	2	2	9
Workshop 2	4	1	1	2	8

### 3.1 Workshop 1 Activities

The first workshop included three co‐design activities. In the first activity, card content was identified using an Affinity Clustering activity [23]. The aim of this activity was to identify and then prioritise what participants wanted to know or thought was important to know in the antenatal, labour and birth, and postnatal period and to organise this information into logical groups. Participants were asked to consider the following question: ‘What do you want to know, or what do you think women and their support people want to know about pregnancy, birthing and their baby's early days?’.

**Photo 1:**
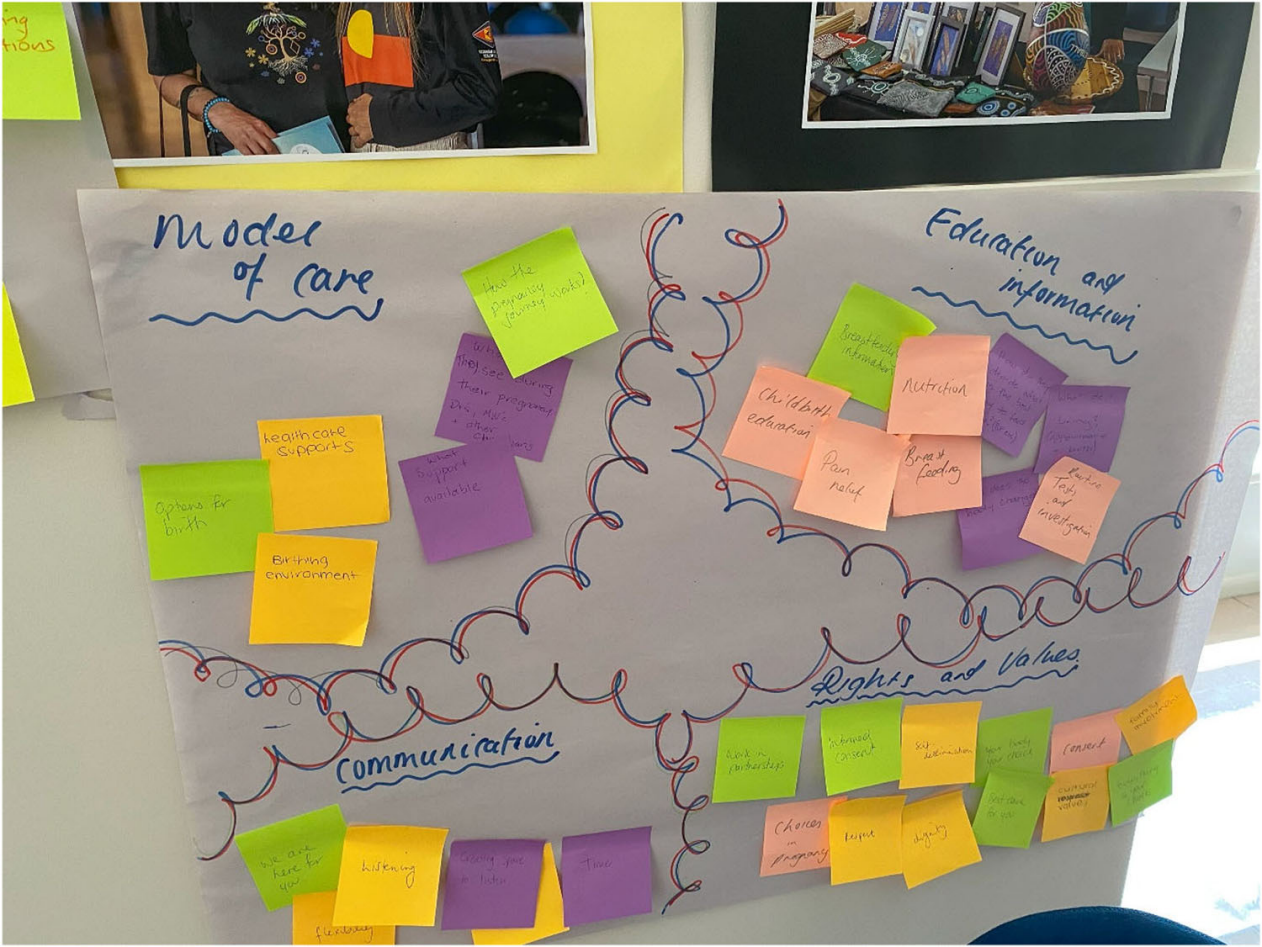
Example of affinity clustering.

Individuals were separated into small groups, and each participant recorded ideas on separate sticky notes. Maternity care clinicians and consumers, and their support people were placed in separate groups. Within the group, one person described and placed an item on the large sheet of paper. Others placed similar items in proximity. This process was repeated until all sticky note suggestions were included on the large sheet of paper. The groups discussed and arranged sticky notes into clusters of similar concepts. The clusters were labelled. Following this, each small group reported back to the larger group about their thoughts and ideas. This enabled thematic patterns to be discussed to build a shared understanding of the possible content. The topics identified by each group are shown in Table 2. An example of Affinity Clustering is illustrated in Photo 1.

**Table 2:** Topics identified by group.

Group	Concepts
1 Women and support people	Family and Support Diet Support Person What If? Self‐Assessment Culture Pain and Assessment If things go wrong Costs Birthing options Support after birth Getting access to the Aboriginal healthcare system
2 Women and support people	Support ‐financial, social Rights Values Education and Information
3 Maternity care clinicians	Communication Model of Care Family Preferences Care Choices
4 Maternity care clinicians	Education Child protection Supports – financial, social

**Photo 2:**
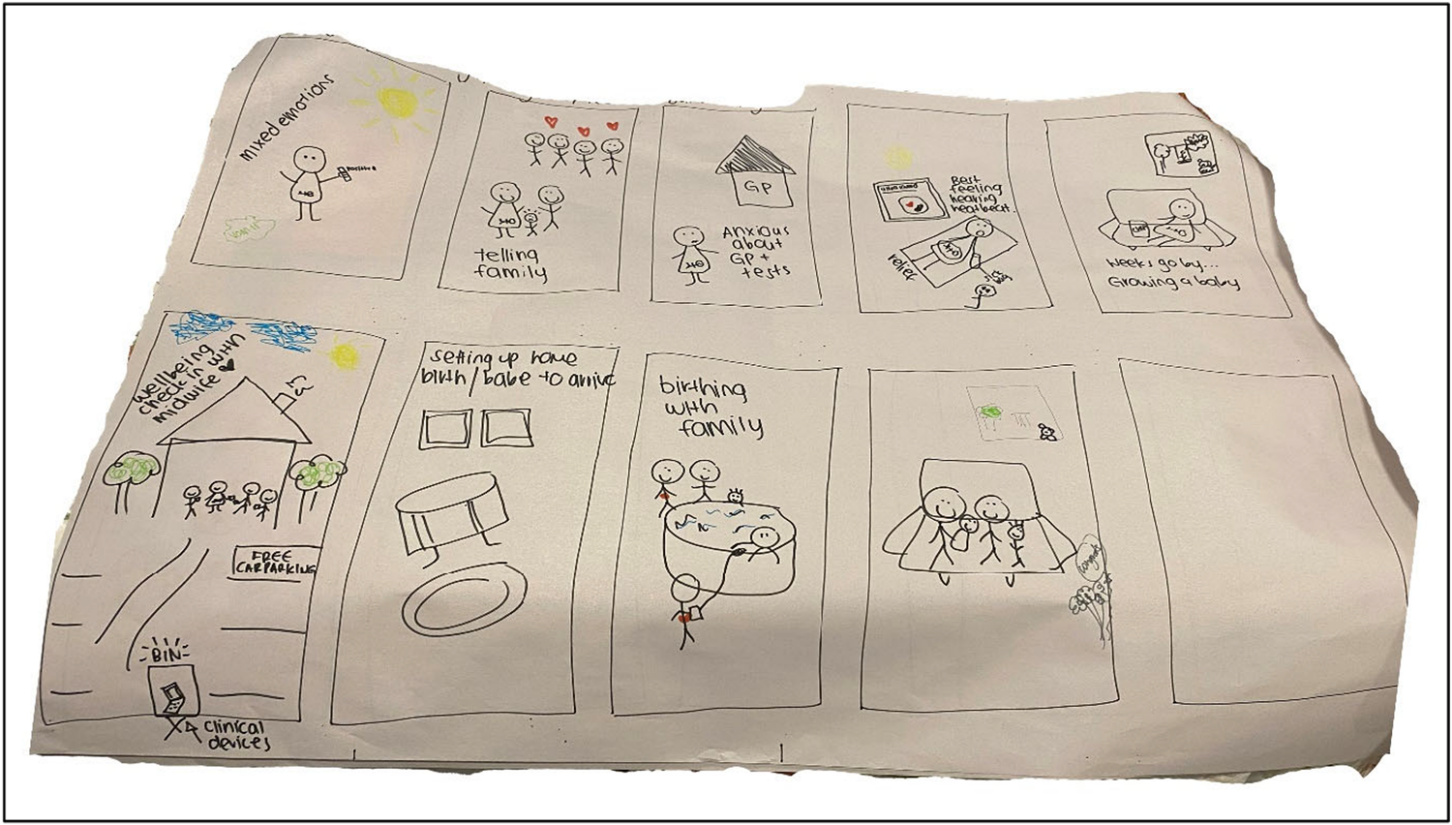
Example of storyboarding.

For the second activity, individuals were randomly allocated into groups, which included a combination of maternity care clinicians, consumers and their support people. The Storyboarding [25] activity aimed to increase understanding of participant's views on ideal culturally safe maternity journey. Participants were asked to draw a series of images and descriptions showing the key elements and interactions in a culturally safe maternity journey for Aboriginal and Torres Strait Islander women. Each group was provided with a large sheet of paper with ten blank rectangles and coloured pencils and pens. They were asked to draw key frames for the scenario, considering the main storyline (beginning, middle and end), the main characters and the setting. In addition, groups were asked to include a descriptive phrase beneath each frame. An example of Storyboarding is illustrated in Photo 2. The Storyboarding activity did not inform the content of the cards, it did, however, assist in developing the Maternity Discussion Cards Quick Reference Guide (see Supporting File S1). The latter details how the Maternity Discussion Cards can be used in the clinical setting.

In the third activity, Rough and Ready Prototyping [26], each group were asked to consider how they would like the maternity discussion cards to look. The purpose of this activity was to ensure the cards would be aesthetically appropriate and usable. Groups were provided basic materials, including paper, scissors and drawing materials. They were given Aboriginal artwork, licenced to the healthcare organisation for consideration. Each small group reported their ideas back to the larger group for discussion.

Following Workshop 1, the research team collated the materials and ideas generated from the workshop. We used an iterative development process that incorporated multiple rounds of review of the workshop outputs by the research team. The concepts and ideas generated in the Affinity Clustering activity informed the content of the cards, for example, ‘supports – financial/social’ translated to discussion cards prompts: Can I talk about my fears and worries?; What cultural supports are available to me?; Who can I talk to about how I am feeling?; Can I access financial counselling?; Who can I talk to if I don't feel safe at home?; What can I do to look after my mental health?; What support will I get from the hospital after my baby is born? The content and appearance of the cards were agreed through consensus by the research team and a total of 67 cards were initially developed. The graphic design team communicated regularly with the principal investigator during the initial design phase to ensure that the cards reflected the workshop data and were practical to use.

### 3.2 Workshop 2

Workshop 2 was held 8 weeks after the first workshop. The aim of Workshop 2 was to refine the content, appearance and usability of the maternity discussion cards. In addition, guidelines for card use in the healthcare setting were developed for consumers and their support persons and maternity care clinicians.

There were three activities in Workshop 2. The purpose of the first activity was to refine the content for the maternity discussion card content. Maternity care clinicians and consumers, and their support people were placed in separate small mixed groups. They remained in these groups for each of the activities. In the first activity, the groups were instructed to briefly consider each printed card and determine the suitability of the card content for maternity care appointments. Groups were instructed to allocate each card into one of four piles: keep, discard, change, or new. Participants were asked to add topics on a blank card to the ‘new’ pile if they considered an important topic was missing.

In the second activity, participants were given an opportunity to provide feedback about the maternity discussion cards regarding the size of the cards and overall appearance, including graphic detail and card quality. They also provided feedback on the wording of the Acknowledgement of Country and description of the artwork. Groups reported that they preferred smaller cards that were easier to carry, and they preferred bold black font. Participants suggested that the cards should be held together with a ring but that the cards could be easily removed from the ring. They also suggested providing a small bag to carry the cards. Groups suggested that the cards should be numbered and categorised into three sections: pregnancy, birth and the baby's early days. Ideas generated are shown in Box 1.

Box 1 **Ideas generated by the groups regarding card design.**

- Aboriginal artwork (Suggested artists; Aboriginal and Torres Strait Islander flags; Aboriginal symbols; photographs).
- Durability (sturdy material, laminated; held together but individual card able to be removed; waterproof).
- Size (able to fit in handbag; discreet; easy to carry).
- Include as part of a Welcome Pack to Midwifery Group Practice.
- Clarity of text appearance and content (small amount of information on card; black print; large font size.
- Provide a link for friends and family to download cards.
- Include blank cards so women/family can add topics that are not already included.
- Categorise cards (number cards, categorise by topic; use colours to denote categories).
- Accessibility (have cards available digitally: store in a dilly bag or box).

The purpose of the final activity, Quick Reference Guide [23], was to develop ‘rules’ or ‘guidelines’ about how the cards might be used in maternity consultations. The groups were instructed to role‐play scenarios, with participants allocated to the roles of a consumer, a maternity care clinician and a scribe. The small groups then reported back to the larger group. The guiding principles for the maternity discussion cards generated during the workshop were summarised by two members of the research team, and this was used to create a short, clear guide summarising key principles and recommendations to aid the implementation of the cards (See Supporting File S1 for Quick Reference Guide).

### 3.3 Final Card Set Design

The research team collated the information from Workshop 2 to inform the design and preparation of the final set of maternity discussion cards. Final discussion card topics were identified based on the following criteria:

- If two (of three) workshop groups decided that a card should be included, it was incorporated.
- Modifications to the wording of the cards were agreed upon by consensus within the research team.
- New suggested topics were included based on the research team's consensus.

**Photo 3:**
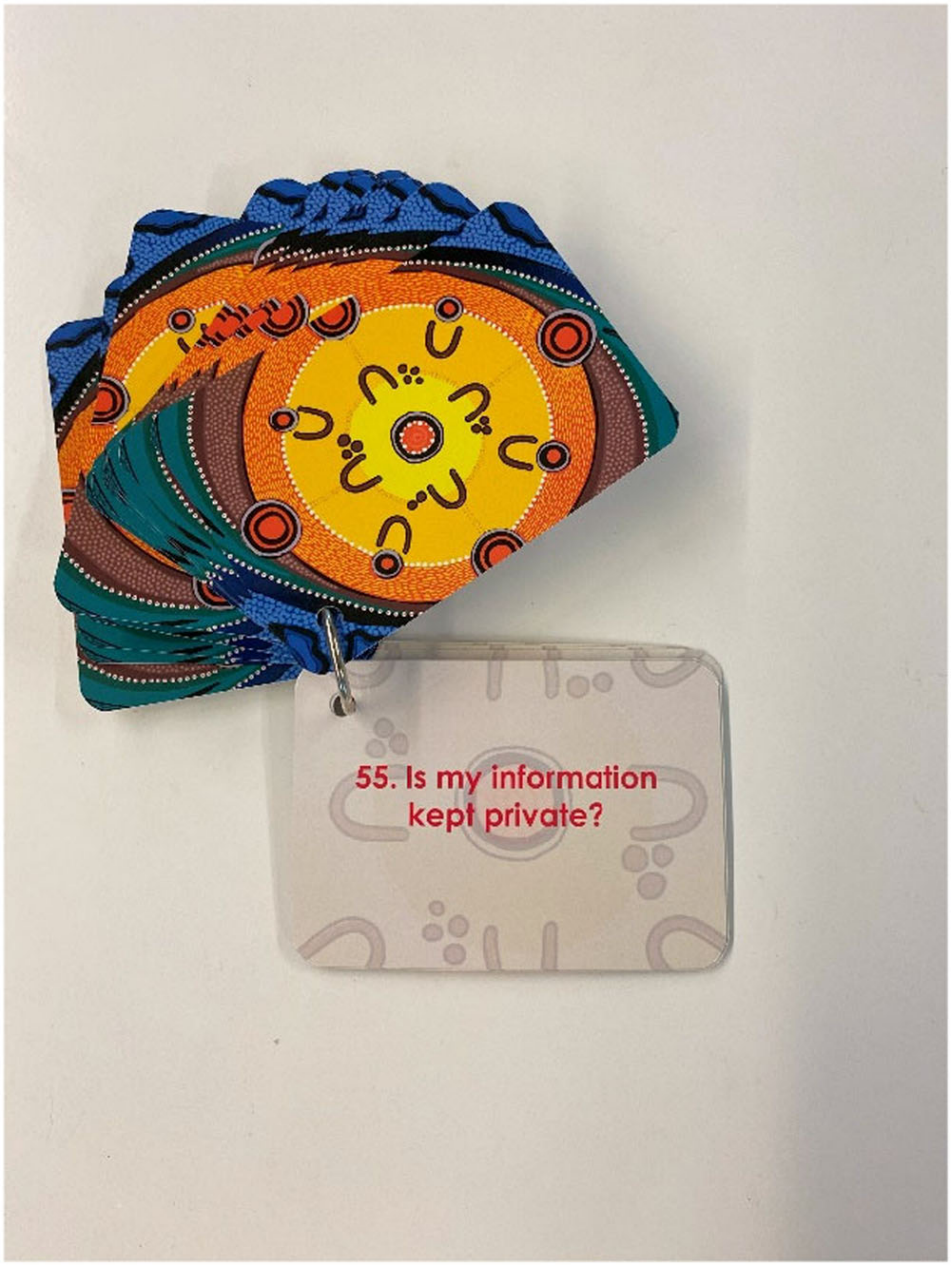
An example of a maternity discussion card.

Each card has a discussion prompt on it (e.g. What baby equipment will I need?, What support is available to help me smoke or vape less?, What are my choices regarding birthing positions?). An example of a card is shown in Photo 3.

Recommendations regarding the design and layout of the maternity discussion cards were carefully considered. The cards were designed with the following design features: cards fastened with a ring for easy removal, categorisation of cards by colour into the three maternity categories: pregnancy, labour and birthing, and early days of parenting, and each card was numbered. In addition, three blank cards were included so that women could write down what they wanted to discuss if a topic was not included. Cards were produced with a durable, waterproof material that could be written on and erased. A participant's suggestion to have the content printed in Braille was rejected based on cost and the plan for the cards to be available on an application with voice technology at a future time. The final set of 56 topic cards and three blank cards, an Acknowledgement of Country and a description of the artwork card and guidelines for card use were established. Fifty sets of the maternity discussion cards were printed for trial use in the dedication of the Aboriginal and Torres Strait Islander MGP in the next phase of the project.

## 4 Discussion

The Maternity Discussion Cards were developed to facilitate shared decision‐making between Aboriginal and Torres Strait Islander women and their partners/support persons, and their maternity care clinicians. This resource parallels the successful culturally appropriate, co‐designed discussion cards developed for use in a palliative care setting [17].

Resources for Aboriginal and Torres Strait Islander peoples must involve end‐users in the processes of planning, delivery and review [27]. The co‐design workshops in this study were interactive, with carefully planned activities that ensured active, enthusiastic and harmonious participation by consumers and maternity care clinicians. The use of central aspects of co‐design [23], such as consultation, meaningful engagement with key stakeholder groups, and collaborative problem solving was essential to the success of the study.

A culturally safe setting, the use of Aboriginal‐owned and led businesses, and ensuring Aboriginal and Torres Strait Islander peoples' voices were key to the planning, delivery, translation and interpretation of the workshop findings laid a platform for the delivery of an ethically sound research project [28].

The maternity discussion cards are envisaged to be a useful tool for women and their support people to self‐determine topics for discussion in their maternity journey in a way that encourages open and honest discussion with their maternity care clinicians. Findings from the co‐design process are encouraging and signal that maternity discussion cards are a resource that can support and facilitate shared decision‐making with women accessing a dedicated Aboriginal and Torres Strait Islander MGP. The maternity discussion cards are consumer‐led and bring together values, goals and preferences, allowing women to decide what information they want to discuss, to reach the most appropriate healthcare decisions for themselves and their family.

While the topics have been developed by Aboriginal and Torres Strait Islander peoples and their maternity care providers in an urban setting, it is hoped that the cards could be tailored to rural and remote settings through a similar co‐design process with the local community. The nature of the cards means that topics that might otherwise be difficult to broach (e.g., child protection, finances, fears and worries) can be more easily raised with the maternity care clinicians, allowing women to request information and support in areas that might be associated with shame or uncertainty [29]. Evidence suggests that maternity care clinicians may avoid difficult conversations due to fear of negative consequences, concerns about imparting shame or stigma and lack of confidence [30]. Also, there are identified practical barriers to having conversations related to time constraints and workload [30]. The maternity discussion cards could be a tool in facilitating these difficult conversations and supporting clinicians to prioritise women's self‐determined topics. The cards will next be piloted in the dedicated MGP and will be evaluated with women, support persons and clinicians to inform further refinement.

The strength of the study lies in the strong Aboriginal and Torres Strait Islander participation, readiness and enthusiasm to co‐design the maternity discussion cards. A study limitation is that the development of the maternity discussion cards was at a single site and relied on participants' experience of one large metropolitan health service or their prior experiences elsewhere. The women's experience of the co‐design activities was not evaluated, and while participants were keen to come to the second workshop and reported they found the experience useful, we did not explore which activities worked best nor how they might be refined. A logical next step for the maternity discussion cards is to further refine the resources and to develop an implementation plan for their integration into clinical practice. This will be the focus of the next stage of the project.

## 5 Conclusions

Together with Aboriginal and Torres Strait Islander women and their support people and maternity care clinicians, we have developed a co‐designed resource to facilitate effective communication and self‐determination in the delivery of maternity care. It is our contention that application of the maternity discussion cards will improve shared decision making in maternity care, leading to improved pregnancy and birth outcomes for Aboriginal and Torres Strait islander women. In the next phase of the project, the resources will be evaluated to determine their acceptability and usefulness for maternity care clinicians and Aboriginal and Torres Strait Islander women in practice.

## Author Contributions

**Tanya Druce:** workshop design, project administration, resources, data management, original manuscript draft preparation, writing, reviewing and editing (lead). **Vidanka Vasilevski:** workshop design, data management, writing, reviewing and editing. **Cara Kennedy:** workshop design, data management, reviewing and editing. **Ann Hallett:** workshop design, resources, workshop organisation, data management, reviewing and editing. **Karah Edwards:** data management, reviewing and editing. **Linda Sweet:** data management, reviewing and editing. **Debra Kerr:** workshop design, data management, writing, reviewing and editing. All authors have read and agreed to the manuscript.

## Ethics Statement

The study received ethics approval on 19th February 2024 from Low‐Risk Human Research Ethics Committees of Western Health (HREC/23/WH/103748) and Deakin HREC.

## Consent

All participants provided written informed consent before participating in the research.

## Conflicts of Interest

The authors declare no conflicts of interest.

## Supporting information

Supplementary material ‐ For review and publication.

## Data Availability

The data that support the findings of this study are available from the corresponding author upon reasonable request.
