# Supplementary material for: Co‐Design of Maternity Discussion Cards for Aboriginal and Torres Strait Islander Women
Source: Health Expect. 2025 Sep 19;28(5):e70437. doi: 10.1111/hex.70437 (PMC12447531; doi:10.1111/hex.70437)
Supplement: Supplementary file 1 — Supplementary material ‐ For review and publication. [file HEX-28-e70437-s001.docx]

**Quick Reference Guide for Use with the Maternity Discussion Cards**

**What are Maternity Discussion Cards?**

Maternity Discussion Cards are a selection of cards with discussion prompts that can be used to guide yarns (or ‘conversations’) with care providers in maternity care about pregnancy, labour and birth, and the early days of parenting. These Maternity Discussion Cards were co-designed with Aboriginal and Torres Strait Islander women, support people and staff from Western Health’s Galinjera Maternity Program and Wilim Berrbang, our Aboriginal Health Unit.

Having a baby is a major life event. Effective communication about issues that are of importance to Aboriginal and Torres Strait Islander women and their support people is a key aspect in self-determination of healthcare needs. The Maternity Discussion Cards should be used following guiding principles:

Working in partnership Right information at the right time

Informed consent Self-determination, trust, and equity

Person-centred care Your body, your choice

Respect and dignity Cultural safety

**What is included in the Maternity Discussion Cards?**

There are a total of 56 discussion cards topics spanning pregnancy, labour and birth, and the early days of parenting. Three additional blank cards can be used to add your own questions if they are not covered by the discussion cards included in the deck. The blank cards are reusable if they are written on with whiteboard markers, as this can be rubbed off. Please avoid using permanent markers. There are also cards that include an Acknowledgement of Country, and a description of the artwork used on the cards.

All cards are marked with coloured dots to identify whether the discussion prompt covers:

- Pregnancy: orange •
- Labour and birth: blue •
- The early days of parenting: brown •
- All stages: teal •

Cards can be selected from any category at any time. The cards are bound by a refastenable ring and numbered, which allows you to remove relevant cards and then return them to the deck once finished.

**How can the Maternity Discussion Cards be used?**

Women are the experts on their bodies, contexts, and healthcare needs. The maternity care needs and expectations are different for different women and their support people. These cards are flexible and can be used in ways that are most appropriate for women, support people, and care providers.

Below are some suggestions on how the cards might be used.

- Ensure women and support people have access to a set of cards, preferably to take home with them.
- The maternity discussion cards can be used at each maternity care appointment, during and after birth, at the hospital or during home visits.
- There are topics that maternity care providers are required to discuss during appointments. The maternity discussion cards sit alongside, rather than replace, the required topics.
- The maternity discussion cards are ideally be used at the beginning of an appointment so there is enough time for a yarn without the need to rush.
- The cards could also be used in a group setting, for example, antenatal classes. Encourage the large group to share their answers to questions raised. Alternatively, create smaller groups and encourage group members to talk about the cards they chose and why.

**Suggestions for using the cards in practice.**

- Ensure that there is time for discussion and that interruptions are unlikely to occur.
- Ask women and support people to select 3-6 cards that are important to them either prior to, in the waiting room, or during the appointment.
- Ask women and their support people to put the cards down on the table in order of importance and prioritise discussions accordingly.
- Ask them if there is anything that is important to them that is not listed on the cards. Use the blank cards to write these down.
- Ask women and families why the card of highest importance was most relevant to them at this time.
- Actively listen. This means being attentive, asking open-ended questions, requesting clarification, and being attuned to and responding to feelings.
- Move on to subsequent cards (as time permits).
- Take note of topics that could not be covered in the time available for follow-up at subsequent appointments.
- Ask women and families to take the cards with them and choose additional topics at their next appointment.
- Follow up on any resources or supports that may benefit women and families as a result of discussions.
- If the woman or support person seems overwhelmed by the number of cards, the cards can be broken into categories (e.g. pregnancy or birth and labour etc.) relevant to the woman’s stage in their pregnancy journey so that there are less cards to consider.
